# Underlying mechanisms of leprosy recurrence in the Western Amazon: a retrospective cohort study

**DOI:** 10.1186/s12879-019-4100-6

**Published:** 2019-05-22

**Authors:** Franciely Gomes Gonçalves, Andréa de Faria Fernandes Belone, Patrícia Sammarco Rosa, Gabriel Zorello Laporta

**Affiliations:** 1 Setor de Pós-graduação, Pesquisa e Inovação, Centro Universitário Saúde ABC, Fundação ABC, Santo André, SP Brazil; 2 Laboratório de Pesquisa e Iniciação Científica, Faculdade Barão do Rio Branco, União Educacional do Norte, Rio Branco, AC Brazil; 3 Serviço Estadual de Dermatologia do Acre (SESACRE), Rio Branco, AC Brazil; 4 Divisão de Pesquisa e Ensino, Instituto Lauro de Souza Lima, Bauru, SP Brazil

**Keywords:** Leprosy, *Mycobacterium leprae*, Drug therapy

## Abstract

**Background:**

The multidrug therapy (MDT) for leprosy treatment adopted by Brazil in the 1990s was important for reducing leprosy in the country; however, recurrent cases remained problematic. Mechanisms involved in leprosy recurrence are heterogeneous and can be sorted into three groups: insufficient therapy, bacillary persistence and new infections. This study aimed to analyse the time interval of leprosy recurrence in relation to the therapeutic scheme in the state of Acre. The hypotheses were as follows: 1) treatments (a) rifampicin, ofloxacin and minocycline (ROM) and (b) dapsone (DDS) have a short leprosy recurrence time, 2) treatments based on MDT have a long leprosy recurrence time, 3) there is a dose-response relationship between MDT and the time interval between leprosy episodes.

**Methods:**

This retrospective cohort study included 201 patients with a second episode of clinical leprosy at the reference centers for leprosy control in the state of Acre. Exposure was the type of therapeutic scheme as follows: 1) ROM, 2) DDS, 3) MDT_0–9 doses_, 4) MDT_10–19 doses_, 5) MDT_20–29 doses_, and 6) MDT_30+ doses_. Outcome was the time interval between release from treatment and a diagnosis of a recurrent leprosy case. Incidence rate ratios and relative risk Poisson regressions adjusted by age and sex were calculated with 95% confidence intervals.

**Results:**

The 201 patients studied during this retrospective follow-up resulted in a total of 224 cases of recurrent leprosy. Incidence rate ratios within this therapeutic scheme were as follows: 3.3 (2.39, 4.2; ROM/MDT_30+_), 1.12 (0.33, 1.92; DDS/MDT_30+_), 2.17 (1.39, 2.94; MDT_0–9_/MDT_30+_), 1.94 (1.13, 2.75; MDT_10–19_/MDT_30+_) and 1.26 (0.47, 2.05; MDT_20–29_/MDT_30+_). Relative risk Poisson regressions showed a protective effect of MDT_30+_ in comparison with ROM (0.22; 0.07, 0.72), MDT_0–9_ (0.42; 0.21, 0.85), and MDT_10–19_ (0.44; 0.21, 0.92). No differences among MDT_30+_ and DDS (0.71; 0.36, 1.41) and MDT_20–29_ (0.76; 0.38, 1.49) were observed.

**Conclusions:**

New infection is an important—yet neglected—mechanism in leprosy recurrence in the state of Acre and can challenge the leprosy elimination plan in Brazil. MDT with few doses might be associated with leprosy recurrence due to insufficient therapy or bacillary persistence.

**Electronic supplementary material:**

The online version of this article (10.1186/s12879-019-4100-6) contains supplementary material, which is available to authorized users.

## Background

In Brazil, the adoption of multidrug therapy (MDT) helped reduce the burden of leprosy [1]. Several strategies have been implemented worldwide, attempting to diagnose leprosy earlier and decrease transmission rates [2]. Thousands of new cases still emerge in this country every year, with a new case detection rate around 12 per 100,000 inhabitants [3]. Ultimately, these new cases could indicate active and widespread foci which maintain the disease in this country.

In the westernmost region of Brazil, a situation can be observed in the state of Acre which demonstrates this dilemma. Despite the achievement of reducing the prevalence of leprosy from 110 to 2 per 10,000 inhabitants, leprosy is still present. An understanding of the mechanisms underlying these recurrences will facilitate implementation of interventions aimed at reducing the bacillary load in exposed populations. In addition, it can further facilitate the interruption of the transmission chain and thus control the disease in the state, according to the recently launched WHO 2016–2020 strategy [4].

Among the mechanisms mentioned in the literature [5–7] as causal factors for leprosy recurrence, the following were frequently reported:

1. Insufficient therapy related to inaccurate classification of the clinical form of the disease at diagnosis, also known as an operational classification error, in which patients with multibacillary leprosy (high infection status) are erroneously classified as paucibacillary (low infection status) and are submitted to inadequate treatment via insufficient doses, and consequently show leprosy recurrence in a short period after treatment completion (< 5 years) [8, 9].
2. Bacillary persistence: even when the clinical diagnosis is correct and the treatment and the number of doses are adequate, it is possible that bacilli of *M. leprae* survive in the host in a dormant phase for a rather long period (5–10 years) within a physiological host–pathogen scenario of low metabolism of the pathogen and host tolerance, further activating its metabolic requirements and multiplying again in the host for unknown reasons, causing clinical manifestations of leprosy in the patient within 10–15 years after release from treatment [10–12].
3. New infection: despite adequate treatment and complete leprosy remission after the cure, it is likely that the patient is re-exposed to *M. leprae* from their social connections (e.g., friends, co-workers, family) in highly endemic areas that contain leprosy transmission hotpots. This scenario increases the risk of pathogen circulation and, consequently, new infection within susceptible patients, manifesting as disease recurrence in a long period after release from treatment (> 15 years) [13].

Herein, leprosy recurrences reported in 2001—2014 in Acre were followed back with medical registries until the first diagnosis in 1950 to carry out our retrospective cohort study. This study aimed to: 1) analyse the time interval between the conclusion of treatment and diagnosis of recurrent disease in relation to the therapeutic scheme, 2) evaluate the incidence rate ratios and relative risk of leprosy recurrence for all therapeutic schemes, and 3) compare leprosy recurrence and new cases among municipalities of Acre. We discuss these results in light of the WHO general objectives for global leprosy elimination.

## Methods

### Study area: the state of Acre

Among the 26 Brazilian states and one federal district—where the capital, Brasilia, is located—Acre is maybe one of the most distant and hard-to-reach because of the logistic issues related to the challenging Amazonian climate and poor transportation infrastructure. Within this state, the access of countryside people to the urban centres of Rio Branco—the state capital—and Cruzeiro do Sul—the second largest city—are possible for only half of the year. The rural citizens’ only options for moving from their homes to the cities are limited by a combination of heavy rainfall and poor roads. This situation causes serious delays in medical access for the treatment of several health issues that affect these rural populations, including leprosy.

**Fig. 1:**
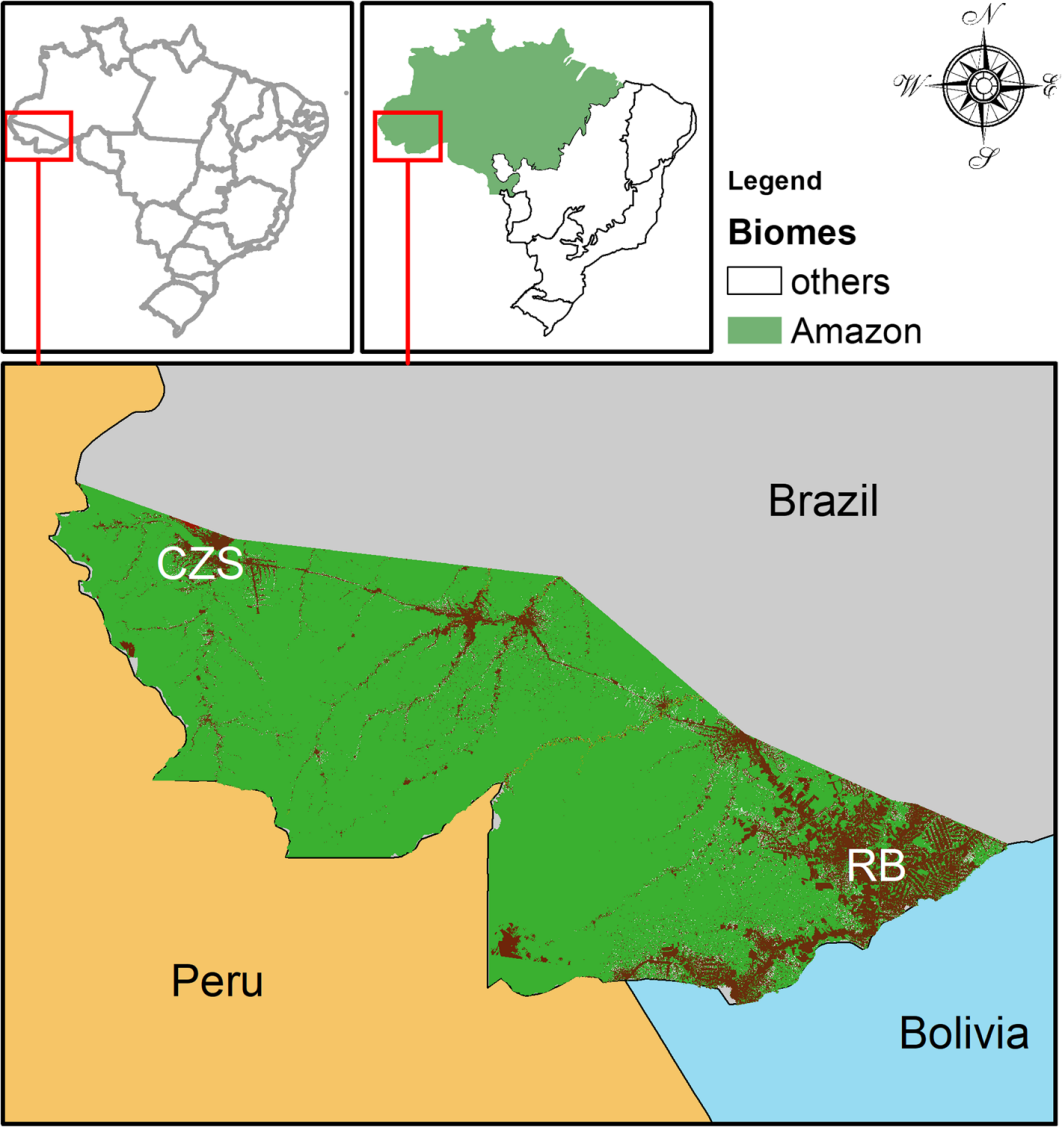
Acre state is the Westernmost among Brazilian states and Amazon biome. It is in the triple frontier border region constituted by Peru, Bolivia and Brazil. Land-use/land-cover data shows that ~ 85% forest cover has been preserved in this state (green areas). Anthropogenic habitats (brown areas) are located along part of the Transamazonian highway system, called BR 364, especially in the urbanized areas of the state capital, Rio Branco (RB) and the second largest city, Cruzeiro do Sul (CZS). This map was exclusively built for this publication in ArcGIS™ v. 10.3.1 with publicly available environmental data in INPE-PRODES and INPE-TerraClass

Acre is in the northwest of Brazil, bordering the state of Amazonas (north), the state of Rondônia (east), Bolivia (south) and Peru (west) (Fig. 1). The average altitude is 200 m above sea level. The climate is hot and humid with two seasons: dry and rainy. The population is about 869,265 inhabitants today. In 2010, there were 733,559 people (2010 Brazilian Census), and of these, 532,279 lived in urban areas and 201,280 in rural areas [14–17]. The human development index is 0.663, which is equivalent to some countries in the Sub-Saharan Africa.

### Study design

This is a retrospective cohort study carried out in the state of Acre. The study included patients from 2001 to 2014 who presented with leprosy recurrence. After the identification of these patients, we performed a search for all medical records—extending to year 1950 in certain cases—associated with each of these patients in both electronic databases and printed registries for data collection on the dates of leprosy diagnosis and release from treatment, therapeutic scheme adopted, and other patient information.

Information regarding the recurrence of leprosy was recorded in the online databases of the National System of Diseases and Notification (SINAN) from 2006 to 2014. From 2001 to 2006, all information was recorded in printed notifications. All these records were provided by the State Service of Sanitary Dermatology, which is the sector in Acre considered to be a reference for the treatment of leprosy. It is in the municipalities of Rio Branco and Cruzeiro do Sul (Fig. 1).

In the present study, 4010 new cases and 201 cases of leprosy recurrence were recorded in the SINAN printed notification sheets of 2006–2014 and 2001–2006. After this first survey, we analysed all medical records related to each patient that were archived and stored from 1954 to 2014 in order to evaluate present and past leprosy recurrences and the type of treatment used in each diagnosis, as well as other demographic information such as sex and birth date.

We were able to build a retrospective follow-up for treated leprosy patients who showed recurrence of the disease at a given time after release from treatment. Exposure considered herein was the type of therapeutic scheme that each patient was exposed to at first diagnosis. Outcome was the time interval between release from treatment and leprosy recurrence.

### Sampling strategy

When the sampling strategy is based on well-known reference centres for the population, any person located within the administrative influence of these centres (i.e., the state of Acre), has the same likelihood of taking part in a given sample. Our sample is represented by all patients enrolled in the treatment of leprosy recurrences between 2001 and 2014 in Acre. Consequently, it is assumed that the detection of leprosy recurrences is random, and all residents of the state have the same probability of being selected for our sample during that period. This further means that a probabilistic sampling strategy was applied.

### Exposure variable: therapeutic scheme

The patients enrolled in this study were exposed to high amplitude therapeutic schemes as follows: 1) ROM, single dose of rifampicin, ofloxacin and minocycline (ROM) for patients with low-infection status, i.e., single lesion paucibacillary leprosy (PB) [18]. 2) DDS, monotherapy with dapsone (DDS), a sulfone used as a treatment before the adoption of multidrug therapy (MDT) [19]. 3) MDT_0–9_, multidrug therapy (MDT) consisting of six doses administered in up to nine months with monthly supervised doses of 600 mg rifampicin + 100 mg dapsone and a self-administered daily dose of 100 mg dapsone, which is recommended for cases with few bacilli that presented with up to five lesions on the skin [20]. We observed that 95% of cases were treated as PB and 5% as high-infection status (i.e., multibacillary leprosy) with 0–9 monthly doses. 4) MDT_10–19_, 5) MDT_20–29_ and 6) MDT_30+_, consisting of MDT applied monthly with 10–19, 20–29, and 30+ doses, respectively. These are treatment schemes utilized for patients with high-infection status or those known as multibacillary leprosy (MB) patients. These multidrug therapies are applied for cases that present with many bacilli and more than five skin lesions, for which are offered monthly supervised doses of 600 mg rifampicin, 300 mg clofazimine and 100 mg dapsone, and a daily dose of 50 mg clofazimine and 100 mg dapsone [21, 22].

The multibacillary scheme of MDT had been utilized in 1982 for a period of at least two years until a negative slit skin smear was observed [21, 22]. In 1993, the WHO recommended the fixed use of 24 monthly doses [23], and only in 2000 did Brazil adopt a regimen of 12 supervised monthly doses [24, 25]. In cases with very high initial bacillary loads, a second round of 12 supervised monthly doses could be prescribed. However, we observed therapeutic schemes with MDT composed of doses other than those mentioned in the literature. Thus, for the purposes of our analysis, we pooled the schemes within a range of monthly doses of MDT.

### Outcome variable

The outcome was the time interval in years between conclusion of treatment and diagnosis of leprosy recurrence. In order to identify leprosy recurrence, we applied the concept from the Brazilian Ministry of Health, which defines a case of recurrence as the one that presents clinical signs and symptoms of the disease [20, 26].

The criteria used to diagnose recurrence are based on: 1) clinical examination with the appearance of new dermato-neurological lesions or exacerbation of old lesions after the treatment is finished that do not respond to the use of corticosteroids; 2) a laboratory test (slit skin smear) that identifies increased number of *M. leprae* in the lesions, ear lobes and/or elbows, or present with solid bacilli characteristic of live organisms; and 3) histopathological examination by skin biopsy [20, 24, 27].

### Data analysis

The time interval was analysed according to therapeutic schemes in the following situations: 1) boxplot, 2) histograms, 3) incidence rate ratio, 4) relative risk of Poisson regressions, and 5) hypothesis testing of medians among groups.

Normality tests were performed with the Shapiro–Wilk (SW) test, and comparisons of medians of the time interval among groups of therapeutic schemes were done by Wilcoxon (W) and Kruskal–Wallis (KW) tests. The significance level adopted for all testing was 5%.

Incidence rate was calculated by the number of leprosy recurrences in a given therapeutic scheme, divided by the person-years of exposure. The incidence rate ratio was calculated by dividing the incidence rate of a given scheme by that of a reference group (i.e., MDT_30+_). A confidence interval of 95% was calculated for the incidence rate ratio.

Poisson regression was applied to cases of leprosy recurrence in three categories of time intervals (0, < 5 years; 1, ≥ 5 < 15 years; 2, ≥ 15 < 30 years). The number of cases in each category was assumed to be random and independent to fit a Poisson regression. The cases were analysed according to independent variables as follows: therapeutic scheme, sex (0, female; 1, male), and age (0, < 40 years;1, ≥ 40 years). Relative risk (RR) and its 95% confidence interval were calculated. Wald test was performed (H_0_: RR = 1).

Finally, a linear regression model was performed to test the linear effect of the incidence of new cases (total and among children/teens) on the leprosy recurrence incidence among municipalities of Acre, 2001–2014. The adopted significance level of each *β*’s marginal t test was 5%. Explanation level of the linear model was estimated by the adjusted R-squared.

### Ethics

This study received approval from the research Ethics Committee of the Hospital das Clínicas do Acre - FUNDHACRE, with the number 1.452.977.

## Results

The 201 patients studied during this retrospective follow-up resulted in a total 224 episodes of leprosy recurrences. The average patient age at these leprosy recurrences was 41.51 years (±16.4). Frequencies of therapeutic schemes adopted were: 1) ROM (14; 6.25%), 2) DDS (45; 20.09%), 3) MDT_0–9_ (72; 32.14%), 4) MDT_10–19_ (37; 16.52%), 5) MDT_20–29_ (49; 21.87%) and 6) MDT_30+_ (7; 3.13%). And, the average time interval between release from treatment and leprosy recurrence diagnosis was 9.13 years (± 6.39, min. = 0.06, max. = 29.18, median = 7.68). This response variable (time interval) does not follow a normal distribution (SW normality test = 0.94, *p* <  0.001).

### Comparing the response variable among therapeutic schemes

**Fig. 2:**
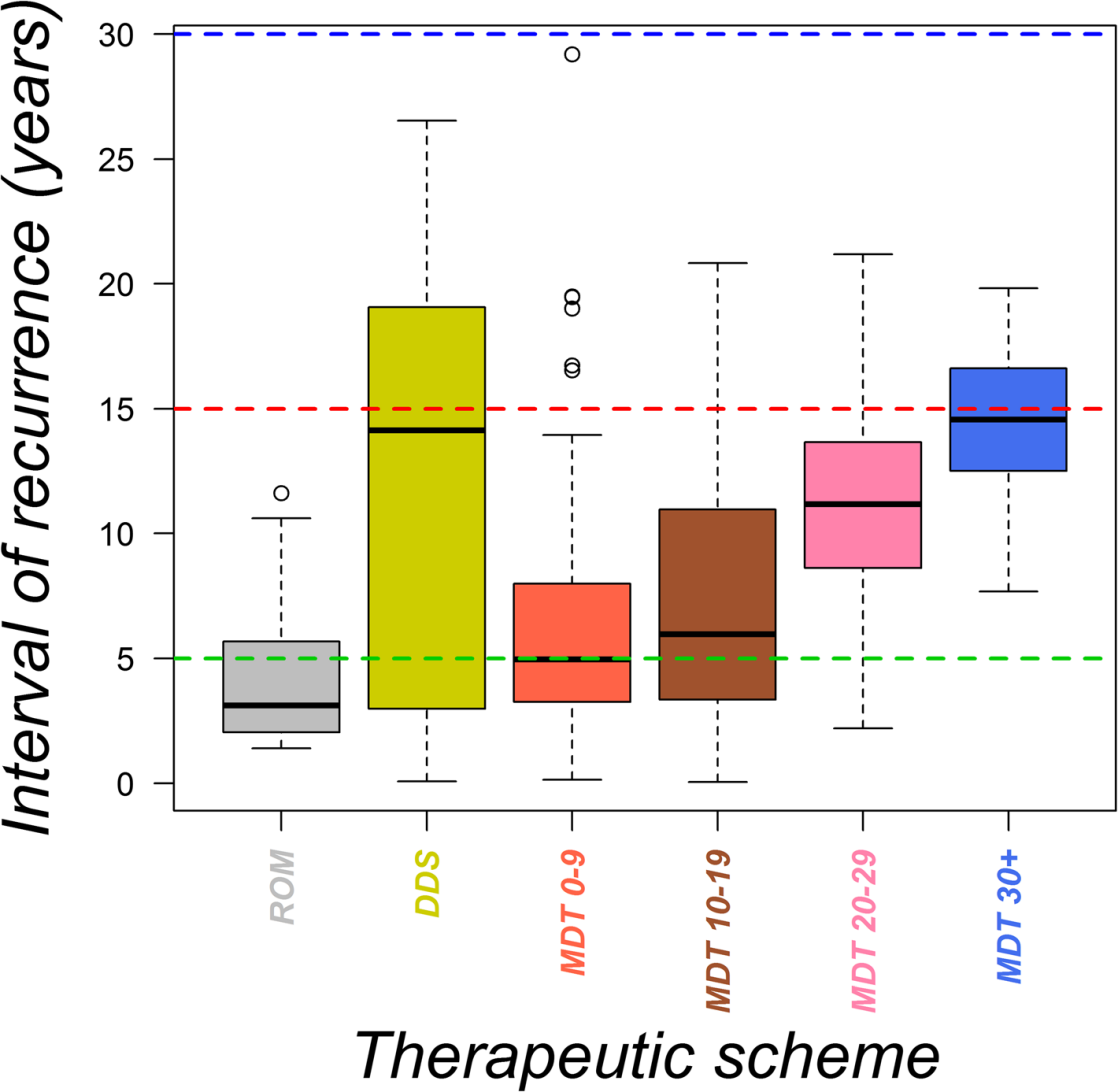
Time interval of leprosy recurrence in years per type of therapeutic scheme adopted. Midlines, upper and lower limits of the box are the median, third and first quartiles, respectively. Ninety-nine % of the data are contained between the upper and lower whiskers. Outliers are represented by open circles

The distributions of intervals of leprosy recurrence were compared among therapeutic schemes using a boxplot (Fig. 2) and were considered statistically different (KW chi-squared = 47.73, df = 5, *p* <  0.001).

**Fig. 3:**
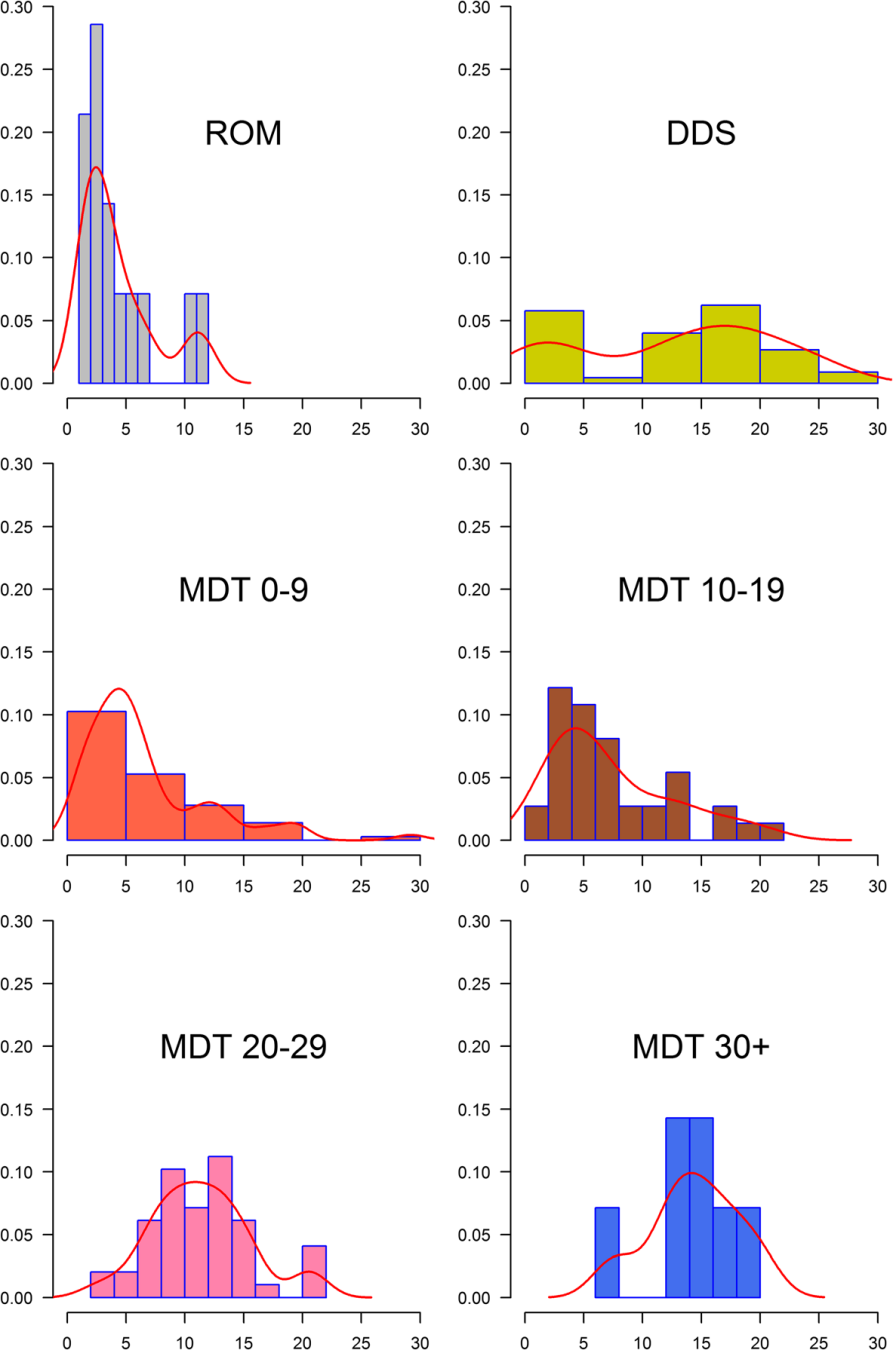
Histograms of interval of leprosy recurrence per therapeutic scheme. Y-axis is the relative frequency (probability) and X-axis is the interval of leprosy recurrence. Red lines are histogram-based density functions

The distributions of intervals of leprosy recurrence per therapeutic scheme were compared using histograms (Fig. 3). The average interval of leprosy recurrence was as follows: ROM, 4.4 years (± 3.3); DDS, 12.8 years (± 8.3); MDT_0–9_, 6.6 years (± 5.4); MDT_10–19_, 7.4 years (± 5.3); MDT_20–29_, 11.4 years (± 4.3); and MDT_30+_, 14.3 years (± 3.9). Moreover, ROM and DDS distributions were bimodal, whereas MDT_0–9_ and MDT_10–19_ were right-skewed distributions, and MDT_20–29_ and MDT_30+_ followed a normal distribution (SW = 0.976, 0.98; *p* = 0.40, 0.97).

Pairwise comparisons among these distributions showed statistically significant results, but not in the following: **ROM – MDT**_**0–9**_ (W = 357.5, *p* = 0.09); **DDS – MDT**_**20–29**_ (W = 1293, *p* = 0.15); **DDS – MDT**_**30+**_ (W = 148, *p* = 0.81); **MDT**_**0–9**_ **– MDT**_**10–19**_ (W = 1192, *p* = 0.37); **MDT**_**20–29**_ **– MDT**_**30+**_ (W = 101, *p* = 0.08).

### Incidence rate association of leprosy recurrence with therapeutic scheme

Numbers and risk association of leprosy recurrence per therapeutic scheme are shown in Table 1. Therapeutic schemes ROM, MDT_0–9_, and MDT_10–19_ had higher incidence rates of leprosy recurrence compared to MDT_30+_. Interestingly, DDS and MDT_20–29_ have the same incidence rate of leprosy recurrence as MDT_30+_ (Table 1).

**Table 1:** Number of leprosy recurrence per therapeutic scheme (N), sum of person-years at risk of leprosy recurrence (py), incidence rate (N/py), incidence rate ratio (IR/IR) and 95% confidence interval of IRR

Therapeutic scheme	N	Person-Year (py)	Incidence rate (IR)	Incidence rate ratio (IRR) (ref: MDT_30+_)	95% CI (IRR)
ROM	14	60.85	0.23	3.3^a^	2.39, 4.2
DDS	45	574.14	0.08	1.12	0.33, 1.92
MDT_0–9_	72	476.45	0.15	2.17^a^	1.39, 2.94
MDT_10–19_	37	273.52	0.14	1.94^a^	1.13, 2.75
MDT_20–29_	49	558.68	0.09	1.26	0.47, 2.05
MDT_30+_	7	100.31	0.07	1	–

^a^Epidemiologically relevant result with higher risk of leprosy recurrence

### Modelling leprosy recurrence as a function of therapeutic scheme adjusted by age and sex

Interval of leprosy recurrence was recoded as an integer with three values (0, < 5 years; 1, ≥ 5 < 15 years; 2, ≥ 15 < 30 years). This recoded variable was modelled in a Poisson regression model as a function of therapeutic scheme (6-level factor) and adjusted by sex (2-level factor) and age (2-level factor) (Table 2).

**Table 2:** Results of multiple Poisson regression models: interval of leprosy recurrence (short, < 5 years, intermediate, ≥5 < 15 yrs., long, ≥15 < 30 yrs.) in function of therapeutic scheme, adjusted by age and sex

Variables	Levels	Relative Risk (adjusted)	95% CI	P (Wald’s test)
Therapeutic scheme (ref: MDT_30+_)	ROM	0.22^a^	0.07, 0.72	0.012
	DDS	0.71	0.36, 1.41	0.322
	MDT_0–9_	0.42^a^	0.21, 0.85	0.016
	MDT_10–19_	0.44^a^	0.21, 0.92	0.03
	MDT_20–29_	0.76	0.38, 1.49	0.423
Sex (ref: female)	1 (male)	0.99	0.71, 1.37	0.941
Age (ref: < 40)	1 (≥40)	1.63	1.19, 2.25	0.003

^a^Statistically significant result according to the assumed significance level (α = 0.05)

Results of Tables 1 and 2 are congruent. ROM, MDT_0–9_ and MDT_10–19_ had higher risks of leprosy recurrence in comparison with MDT_30+_. On the other hand, DDS and MDT_20–29_ had the same risk of leprosy recurrence as MDT_30+_.

### Underlying mechanisms in leprosy recurrence

Here, we compared the median time interval for leprosy recurrence in each category of time interval according to therapeutic scheme (Table 3). The categories of time interval were chosen based on the most likely underlying hypothetical mechanisms: < 5 years, insufficient therapy due to operational classification error; ≥ 5 < 15 years, bacillary persistence and resistance; ≥ 15 years, new infection.

**Table 3:** Comparison of the median of time interval for leprosy recurrence in each category of time interval by therapeutic scheme

Time interval categories	ROM	DDS	MDT 0–9	MDT 10–19	MDT 20–29	MDT 30+	KW test (p)
≥ 15 years	–	19.06	19.24	17.81	16.12	17.58	4.15 (0.38)
≥ 5 < 15 years	8.8	11.7	7.2	8.4	10.5	12.5	14.01 (0.016)^a^
< 5 years	2.42	1.65	3.3	3.3	2.8	–	9.55 (0.049)^a^

^a^Results of the Kruskal-Wallis rank sum test. Statistically significant results (*p* <  0.05) showed differences of the medians among groups of therapeutic scheme

### Linear models of leprosy recurrence as a function of new cases

Coefficients of leprosy recurrence cases (100,000 ppl.), total new cases (10,000 ppl.), and new cases in children and teens (< 15 years old; 100,000 ppl.) from 2001 to 2014 were analysed in a linear regression model, with municipalities of Acre as the units of analyses (Table 4).

**Table 4:** Linear regression models of leprosy recurrence in function of new cases in the municipalities of Acre state, 2001–2014

Response variable	Explanatory variables	Linear model	*β*	P	R^2^
Recurrence^a^	New cases^b^	lm1	0.19	< 0.01^*^	0.33
Recurrence	New cases < 15 years-old^a^	lm2	3.021	< 0.001^*^	0.44
Recurrence	New cases	lm3	0.4	< 0.05^*^	0.53
	New cases < 15 years-old		2.41	< 0.01^*^	

^a^Coefficient per 100,000 persons

^b^Coefficient per 100,000 persons

^*^Statistically significant results of the marginal t test of each *β* (*p* < 0.05)

Multiple linear model lm3 had the best fitting (R^2^ = 0.53), and its residuals followed a normal distribution (SW = 0.97, *p* = 0.73). New cases’ *β* (0.4) indicated overfitting due to its higher value comparing to lm1. On the other hand, new cases for < 15 year-olds *β* (2.41) showed 20% adjustment compared to lm2. As a conclusion, for each new case in < 15 year-olds there will be 2.41 cases of leprosy recurrence in the municipalities of Acre.

## Discussion

The implementation of multidrug therapy (MDT) for leprosy control has been important for the reduction of the overall burden of disease in the world. However, the emergence of new cases every year in endemic countries is of great concern. In 2015, out of the 210,758 new cases in the world, India occupied first place with 60.1% of the cases, and Brazil is ranked second with 12.5% [4]. The incidence in children suggests active transmission of the disease in approximately 100 countries [28]. In the westernmost state of Amazonia, the situation is similar. Acre occupies the third place in the number of new cases in Brazil and contributed to the occurrence of 125 new cases; in 2017, 10 of these cases were in children/teens under 15 years of age [3]. This situation points to the need for new global and local preventive measures that facilitate the control of the disease in scenarios of hotspot *M. leprae* transmission. Cases of leprosy recurrence in patients that were considered cured appear in the surveillance system and can be erroneously related to factors other than new infections. Among the strategies for controlling and monitoring the success achieved by leprosy control programs is the identification of the underlying mechanisms involved in the recurrence of the disease in the target population.

### Historical appraisal

The implementation of the multidrug therapy adopted by Brazil in the 1990s has had an important impact on the reduction of the leprosy endemic in the country. This created a situation that positively modified the leprosy epidemiology, with a remarkable trend toward a decline in prevalence among Brazilian populations. However, leprosy recurrence could not be avoided under this MDT intervention. Developing new concepts or perspectives that favour the comprehension and further implementation of novel control interventions can therefore interrupt the transmission chain and consequently reduce leprosy magnitude [29, 30].

### Conceptual model: rationale and proposition

One of the novelties of the present study is the interpretation of the results while considering the challenging agenda for the elimination of leprosy in Brazil. In this context, we considered heterogeneous underlying mechanisms involved in leprosy recurrence. In order to identify these mechanisms with the available data, we assumed that each one had a characteristic length of time from release from treatment to leprosy recurrence. In our proposed evaluation, we consider the time interval under 5 years as having the underlying mechanism of insufficient therapy based on the operational classification error at the time of the first diagnosis. In such a case, multibacillary leprosy cases classified as paucibacillary receive treatment with insufficient doses, which might increase the likelihood of recurrence [31]. When the time interval is ≥5 < 15 years, the underlying mechanism is very likely bacillary persistence, which refers to the adaptive capacity of *M. leprae* bacilli to remain inactive under conditions of low metabolism during treatment and reacquire its active metabolic form sometime after release from treatment, causing new signs of disease activity [32]. When the time interval is ≥15 years, the underlying mechanism favours new infection. Although this mechanism is hard to scientifically demonstrate, we considered it in our study using logical deduction. Leprosy recurrence cases that present with long time intervals between the first and second treatments are mainly found in those patients that remain continuously exposed to high bacillary contact in local transmission hotspots [33–35].

### Underlying mechanisms in leprosy recurrence

In our study, we tested the dose-response effect of MDT. Accordingly, we expected that the lowest dosage (i.e., MDT_0–9_) would imply a greater probability of recurrence in a shorter period (under 5 years). This relationship was observed mainly in patients submitted to the following schemes: MDT_0–9_ and MDT_10–19_. Conversely, higher dosage (i.e., MDT_30+_) can lower the likelihood of recurrence. This was associated with a longer time interval between release from treatment and recurrence, as shown for MDT_20–29_ and MDT_30+_. In agreement with the literature and our conceptual model, we interpreted these results as a consequence of the mechanism of therapeutic insufficiency for the patients erroneously classified as paucibacillary leprosy, such as those in the group of MDT_0–9_, which consequently led to inadequate treatment and disease recurrence [36–38].

Accordingly, patients submitted to ROM and DDS schemes presented a bimodal distribution for the relevant time intervals. Their first peaks were equivalent and corresponded to a high probability of therapeutic insufficiency. The MDT_0–9_ scheme also presented a greater predominance in the time interval under 5 years, which is related to therapeutic insufficiency according to the Brazilian Ministry of Health.

A higher incidence rate of leprosy recurrence was observed for the cases submitted to ROM, MDT_0–9_ and MDT_10–19_, compared to MDT_30+_. However, there is no statistical difference between incidence rates of leprosy recurrence of the DDS and MDT_30+_ schemes. We emphasize that this similarity between these diametrically opposed therapeutic schemes had contributions from mechanisms other than the insufficient therapy mechanism. Particularly, the MDT_30+_ scheme showed a protective effect compared to the ROM, MDT_0–9_ and MDT_10–19_ schemes for leprosy recurrence.

In addition to the insufficient therapy mechanism, we observed the likelihood of the mechanism of bacillary persistence. For instance, there is a representative number of cases (11%, 23/224) that showed leprosy recurrence associated with a time interval of 10.5 and 12.5 years. Balagon et al. found in their study a peak of leprosy recurrence between 11 and 12 years, suggesting that this peak is related to the activation of dormant organisms that did not undergo effective MDT [39, 40]. Additionally, for recurrent cases that occurred between 10 and 15 years—especially those who had more than one recurrence episode—we emphasize the importance of investigating bacterial resistance through molecular analysis. For instance, we have currently been testing contacts of new leprosy cases for the possibility of infection by resistant bacteria. So far, this mechanism does not seem to be widespread in Acre (data not shown/not published).

One important finding of our study is that patients given more prolonged treatments (e.g., MDT_20–29_ and MDT_30+_) have a lower risk of leprosy recurrence. This obviously challenges recent studies demonstrating the ubiquitous efficacy of the newly proposed therapeutic scheme known as the uniform multidrug therapy (MDT-U). MDT-U is a shorter dosage scheme for the treatment of MB leprosy and contains the addition of one drug to PB leprosy [41]. Of course, this is a matter of intense debate, and it is not within the scope of the current study.

Another important finding is that the mechanism of new infection in leprosy recurrence is both epidemiologically important and neglected. For instance, the second peak of the time interval in patients that had DDS for a therapeutic scheme is emblematic. Almost 50% (22/45) of the patients in the DDS therapeutic scheme showed leprosy recurrence in 15 or more years after the cure. This is in congruence with the high and positive linear effect of the incidence of new cases—especially in children under 15—on incidence of recurrent leprosy cases in the municipalities of Acre. The mechanism of new infection is therefore one of the causes of leprosy recurrence [42–46].

### Limitations

We have not applied whole genome sequencing to distinguish between recurrence and reinfection with a new strain of *M. leprae* [33].

## Conclusions

Given that a therapeutic scheme (DDS) classically recognized as flawed showed a similar time interval between release from treatment and the leprosy recurrence episode in comparison to the most efficient therapeutic scheme (multidrug therapy with 30+ monthly doses), we conclude that a random mechanism is causing recurrence of leprosy in the state of Acre. This random mechanism was logically deducted as new infection, supported by endemic foci of leprosy transmission.

Because there is a strong dose-response relationship among therapeutic schemes based on multidrug therapy, we conclude that fewer doses of MDT could be associated with a high likelihood of insufficient therapy and/or bacillary persistence in recurrence of leprosy. This further challenged the novel proposal of a uniform multidrug therapy with 6 monthly doses.

## Additional files

Additional file 1: Retrospective cohort study on leprosy recurrence in Acre State: raw data, glossary, patients and recurrences. Spreadsheet 1, raw data: data retrieved from medical prontuaries. Spreadsheet 2, glossary: complementary information on the acronyms or abbreviations present in the raw data spreadsheet. Spreadsheet 3, patients: age, sex, therapeutic scheme and the time interval between release from treatment and a new diagnosis for each patient with a second episode of leprosy. Spreadsheet 4, recurrences: age, sex, therapeutic scheme and the time interval between release from treatment and a new diagnosis for each leprosy recurrent case. (XLSX 76 kb)

Additional file 2: New cases and recurrent cases of leprosy in Acre municipalities. Spreadsheet 1, new cases of leprosy per municipalities, 2001–2014. Spreadsheet 2, new cases of leprosy in under of 15 years old per municipalities, 2001–2014. Spreadsheet 3, recurrent cases of leprosy per municipalities, 2001–2014. Spreadsheet 4, Census and estimated population per municipalities, 2001–2014. (XLS 50 kb)

## Abbreviations

DDS: monotherapy with dapsone
KW: Kruskal-Wallis test
MB: multibacillary leprosy
MDT: multidrug therapy
MDT_0–9_: up to nine monthly doses of MDT
MDT_10–19_: up to nineteen monthly doses of MDT
MDT_20–29_: up to twenty-nine monthly doses of MDT
MDT_30+_: thirty or more monthly doses of MDT
MDT-U: uniform multidrug therapy
PB: paucibacillary leprosy
ROM: single dose of rifampicin, ofloxacin and minocycline
RR: relative risk
SINAN: National System of Diseases and Notification
SW: Shapiro-Wilk test
WHO: World Health Organization
